# High-temperature differences in plasmonic broadband absorber on PET and Si substrates

**DOI:** 10.1038/s41598-020-70268-8

**Published:** 2020-08-06

**Authors:** Jin Hee Kim, Sung-Gyu Lee, Teun-Teun Kim, Taewoo Ha, Sang Hyup Lee, Ji-Hee Kim, Young Hee Lee

**Affiliations:** 1 Department of Applied Physics, Institute of Natural Science, Kyung Hee University, Yong-in, Gyeong-gi 17104 Republic of Korea; 2 Center for Integrated Nanostructure Physics, Institute for Basic Science (IBS), Suwon, 16419 Republic of Korea; 3 Department of Energy Science, Sungkyunkwan University, Suwon, 16419 Republic of Korea

**Keywords:** Thermoelectrics, Nanophotonics and plasmonics

## Abstract

The characteristics of a plasmonic resonator with a metal–dielectric–metal structure is influenced by the size, shape, and spacing of the nanostructure. The plasmonic resonators can be used in various applications such as color filters, light emitting diodes, photodetectors, and broadband absorbers. In particular, broadband absorbers are widely used in thermophotovoltaics and thermoelectrics. To achieve a higher photothermal conversion efficiency, it is important to provoke a larger temperature difference in the absorber. The absorption and thermal conductance of the absorber has a great impact on the temperature difference, but in order to further improve the temperature difference of the absorber, the thermal conductivity of the substrate should be considered carefully. In this study, we designed Cr/SiO_2_/Cr absorbers on different substrates, i.e., polyethylene terephthalate (PET) and silicon. Although their optical properties do not change significantly, the temperature difference of the absorber on the PET substrate is considerably higher than that on the Si substrate under laser illumination, i.e., 164 K for ΔT_PET_ and 3.7 K for ΔT_Si_, respectively. This is attributed to the thermal conductance of the substrate materials, which is confirmed by the thermal relaxation time. Moreover, the Seebeck coefficient of graphene on the absorber, 9.8 μV/K, is obtained by photothermoelectrics. The proposed Cr/SiO_2_/Cr structure provides a clear scheme to achieve high performance in photothermoelectric devices.

## Introduction

Plasmonic nanostructures, including metallic and dielectric materials, yield various interesting optical phenomena such as resonant cavities, waveguides, nanoantennas, and metasurfaces^1–3^. A metal–dielectric–metal (MDM) resonator is one of the simplest and most fascinating plasmonic nanostructures, which is based on the Fabry–Pérot (F–P) resonance and comprises two reflective metal layers separated by an optically transparent dielectric material^4^. The optical properties of an MDM resonator can be controlled by the size, shape, and periodicity of the nanostructures, because the eigenfrequency of the F–P resonance is determined using the distance of two reflectors^2–4^. The optical properties controllable by the artificial nanostructure of MDM resonators enable us to employ these resonators as color filters^2,5,6^, colored solar cells^7^, and solar absorbers for thermophotovoltaics^8^ and thermoelectrics^9,10^. Moreover, electrically tunable optical properties of the MDM structure enable new applications such as high-resolution displays, optical communications, and color-tunable windows^11,12^. MDM layered structures by depositing metal and dielectric layers, are advantageous for commercialization because they can be easily applied to large size films and lithography-free fabrications^3,13^ When using lossy metals, such as Cr, Ni, W, and Ti, the MDM structure can absorb a broadband light spectrum with a high absorption intensity exceeding 95% and wavelength ranges from 400–800 nm^2,13,14^. In the broadband MDM absorber, an upper thin metal layer is used for absorption, an intermediate transparent layer is used as the dielectric material (Al_2_O_3_, SiO_2_, CaF_2_, BK_7_, KCl, and MgF_2_), and a lower thick metal layer is used for reflection^14^.

The efficiency of thermoelectric conversion strongly depends on the thermoelectric figure of merit (ZT) as well as the temperature difference (ΔT). The temperature difference of a broadband MDM absorber can be enhanced via heat absorption under light illumination, thereby improving the device performance in thermoelectric applications. For instance, the thermoelectric conversion efficiency (η) at ΔT = 200 K (ZT = 1) is 8.2%, which is significantly higher than η = 4.8%, which is obtained at ΔT = 100 K^15^. The relationship between heat (Q) and temperature difference (ΔT) is expressed as the following relation: $Q={C}_{P}m\Delta T$, where ${C}_{P}$ and *m* are the specific heat and mass, respectively^16^. The heat flow ($\overrightarrow{Q}$) is intimately related to the thermal conductivity ($\kappa$) based on the Fourier law: $\overrightarrow{Q}=-\kappa \overrightarrow{\nabla }T$, where $\overrightarrow{\nabla }T$ is the temperature gradient^17^. A large ΔT is achievable because the broadband MDM absorber has a low mass owing to the nanoscale thickness of the metal layers. However, for a high $\overrightarrow{\nabla }T$, the thermal conductivity of the absorber should be carefully considered because the heat generated through light absorption in the top metal layer can be easily transferred to the substrate.

In this study, we demonstrate a Cr/SiO_2_/Cr absorber with polyethylene terephthalate (PET) and Si substrates to determine the effect of substrate thermal conductivity for efficient photothermal conversion. Regardless of the type of substrate, the simple Cr/SiO_2_/Cr layered structure absorbs light with high absorption (~ 97%), over visible wavelengths ranging from 450–800 nm^13^. However, the absorber on the PET with a low thermal conductivity, κ = 0.15 W/mK^18^, shows ~ 40 times higher temperature difference, compared to that on the Si substrate (bulk Si: κ = 40–150 W/mK^19,20^). Furthermore, under the large temperature difference, we obtained the Seebeck coefficient of monolayer graphene on the absorber with the PET to be approximately 9.8 μV/K.

## Results and discussion

### Schematic and optical property of the absorber

**Figure 1:**
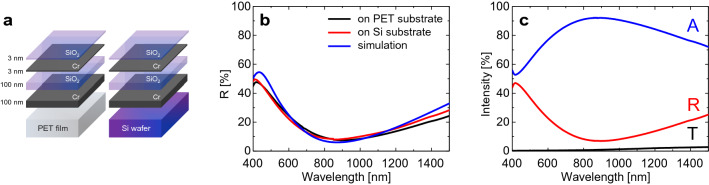
Configuration schematics and performance of the Cr/SiO_2_/Cr absorber. (**a**) Schematic of the Cr/SiO_2_/Cr absorber (**b**) measured and simulated reflectance (R%) of the Cr/SiO_2_/Cr absorber on PET and Si substrates, (**c**) Measured absorptance (A%), reflectance (R%), and transmittance (T%) of the Cr/SiO_2_/Cr absorber on a PET substrate.

Schematics of the Cr/SiO_2_/Cr absorber on the PET and Si substrates are presented in Fig. 1a. The Cr/SiO_2_/Cr absorber comprises a top Cr layer (3 nm), middle transparent SiO_2_ layer (100 nm), and bottom reflection Cr layer (100 nm), with a SiO_2_ layer (3 nm) on the top Cr layer to prevent oxidation^13^. The Cr and SiO_2_ layers were evaporated using an electron beam evaporator on the PET and Si substrates. The measured reflectance spectra of the Cr/SiO_2_/Cr absorber on the PET and Si substrates indicate that the optical properties did not change significantly with the change in the substrate, as shown in Fig. 1b. Furthermore, using finite element method (FEM) simulations, the reflectance of the Cr/SiO_2_/Cr structure was calculated. The difference between the measured and calculated reflectance was primarily caused by experimental errors, such as the thickness and roughness of the Cr and SiO_2_ layers. Figure 1c illustrates the absorptance (A%), reflectance (R%), and transmittance (T%) of the Cr/SiO_2_/Cr absorber on the PET substrate. Based on the measured reflectance (R%) and transmittance (T%), the absorptance (A%) was obtained using the simple relation: A + R + T = 100 (%)^13,14^. The maximum absorptance of the Cr/SiO_2_/Cr absorber on the PET substrate was 92% near a wavelength of 870 nm, which response may show good absorptance performance in angled illumination as well^2,13^.

### Temperature of the absorber

**Figure 2:**
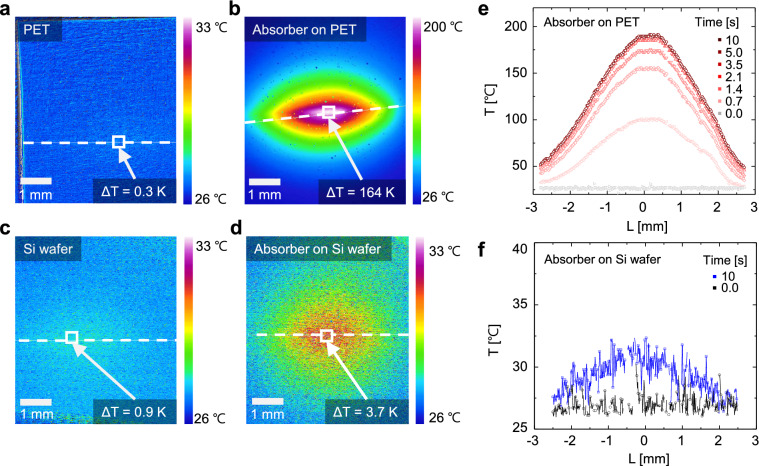
Measured temperature of the absorbers under illumination. Thermal images of (**a**) the PET substrate, (**b**) the Cr/SiO_2_/Cr absorber on the PET substrate, (**c**) the Si substrate, and (**d**) the Cr/SiO_2_/Cr absorber on the Si substrate, under laser illumination (906 nm, 70 mW, 10 s). Temperatures of the Cr/SiO_2_/Cr absorber on the (**e**) PET and (**f**) Si substrates with respect to the positions and laser illumination time at the white line in the thermal images.

The photothermal performances of the Cr/SiO_2_/Cr absorber on the PET substrate, Cr/SiO_2_/Cr absorber on the Si substrate, and bare PET and Si substrates were investigated using an infrared camera system (InfraScope) under laser illumination in the ambient atmosphere. Figure 2a–d show the thermal images under a 906-nm laser illumination with a laser power of 70 mW. The average temperatures of the white-square-marked areas in the thermal images differed vastly with respect to the substrates. The average temperature of the Cr/SiO_2_/Cr absorber on the PET substrate increased significantly from 26.7 °C to 190.7 °C (ΔT = 164 K), as compared with the Cr/SiO_2_/Cr absorber on the Si substrate (ΔT = 3.7 K), bare PET (ΔT = 0.3 K), and Si (ΔT = 0.9 K). In particular, as shown in Fig. 2e, the temperatures of the Cr/SiO_2_/Cr absorber on the PET substrate differed significantly in comparison to that on the Si substrate, even though they had identical structures with similar absorptance.

### Photothermal performance

**Figure 3:**
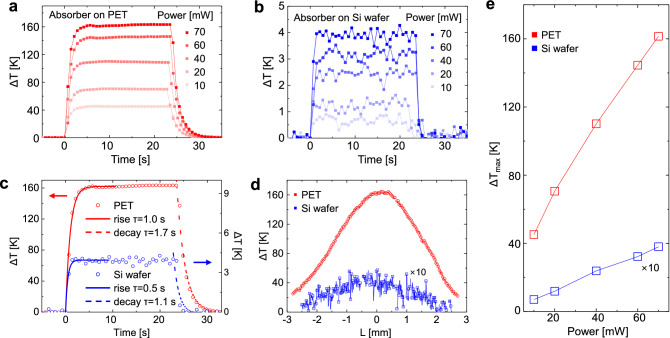
Measured photothermal performance of the absorbers under illumination; time-dependent temperature difference of the absorber on the (**a**) PET and (**b**) Si substrates under different laser powers. (**c**) Thermal relaxation curve under laser illumination (906 nm, 70 mW). (**d**) Temperature difference of the absorber on the PET and Si substrates with respect to the positions under laser illumination (906 nm, 70 mW, 10 s). (**e**) Temperature difference as a function of laser power.

Figure 3a,b present the time-dependent temperature differences (ΔT) of the Cr/SiO_2_/Cr absorber on the PET and Si substrates, under different laser powers. The relaxation behavior of the temperature differences appeared during laser on and off. As shown in Fig. 3c, the thermal relaxation times of the absorbers were calculated via the fitting method, using the exponential rise (solid line) and decay (dashed line in Fig. 3c), ΔT = A exp(1 − t/τ), and ΔT = A exp(t/τ), where A and τ are a constant and the relaxation time, respectively^21,22^. The result shows that the thermal relaxation time of the Cr/SiO_2_/Cr absorber on the PET substrate (τ_rise_ = 1.0 s, τ_decay_ = 1.7 s) is longer than that of the Cr/SiO_2_/Cr absorber on the Si substrate (τ_rise_ = 0.5 s, τ_decay_ = 1.1 s). The thermal relaxation time (τ) is related to the heat capacity (C) and thermal conductance (K) of the sample according to the relation: τ = C/K^16,21^. The long thermal relaxation time (τ) of the Cr/SiO_2_/Cr absorber on the PET substrate was affected by the low thermal conductivity of PET (0.15 W/mK^18^), and the short thermal relaxation time (τ) of the Cr/SiO_2_/Cr absorber on the Si substrate was a result of the high thermal conductivity of the bulk Si (40–150 W/mK^19,20^). This difference in the thermal relaxation times of the absorbers indicates that the substrate affected the photothermal performance due to heat transfer. Figure 3d shows that the maximum temperature differences of the Cr/SiO_2_/Cr absorber on the PET and Si substrates are approximately 160 K and 4 K under a 906 nm laser illumination (70 mW, 10 s), respectively. As shown in Fig. 3e, the maximum temperature differences of the absorbers increase monotonously with increasing laser power. The maximum temperature difference of the Cr/SiO_2_/Cr absorber on the PET substrate was significantly higher than that of the Cr/SiO_2_/Cr absorber on the Si substrate.

### Thermoelectricity in graphene

**Figure 4:**
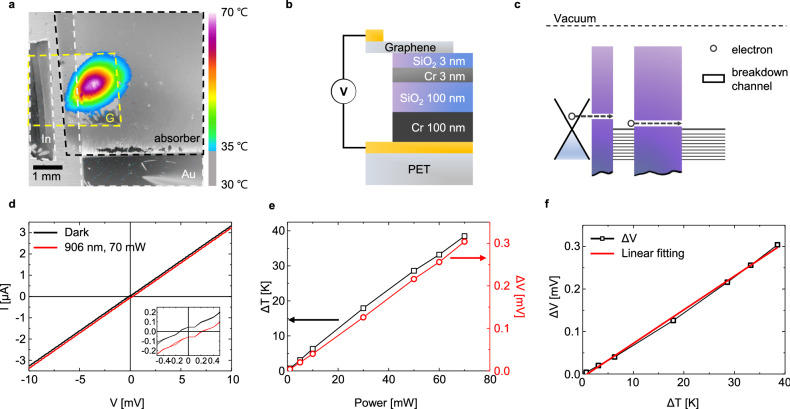
Seebeck coefficient measurement of single-layer graphene. (**a**) Optical and thermal image, (**b**) schematics, and (**c**) energy band diagram of single-layer graphene on the Cr/SiO_2_/Cr absorber using the PET substrate, under laser illumination (906 nm, 70 mW, 10 s). (**d**) Current–voltage (I–V) curves in the dark and under laser illumination. (**e**) Dependence of photovoltage and temperature difference on laser power. (**f**) Voltage difference versus temperature difference for the total Seebeck coefficient.

The Seebeck coefficient of single-layer graphene was measured using the photothermoelectric effect, which is based on the thermoelectric effect with a temperature gradient due to light absorption^23,24^. Figure 4a shows the optical and thermal images of the monolayer graphene on the Cr/SiO_2_/Cr absorber with a PET substrate, under laser illumination (906 nm, 70 mW, 10 s). The Cr/SiO_2_/Cr absorber was evaporated using an electron beam evaporator on a Au-coated PET substrate, as shown in Fig. 4a,b. Bulk indium was used to establish an electrical contact with the other electrode. Figure 4c shows the energy band diagram of the graphene (work function: 4.5 eV^25^) on the absorber (work function of Cr: 4.5 eV^26^, electron affinity of SiO_2_: 0.9 eV, band gap energy of SiO_2_: 9 eV^27^) with the electrodes (work function of Au: 5.3 eV, In: 4.1 eV^26^). The I–V curve was obtained by the leakage current of the deposited SiO_2_ layer (see Fig. 4d). The I–V curve exhibits a linear behavior under both dark and illumination conditions (906 nm, 70 mW, 10 s). The I–V curve is shifted under the illumination condition, as compared with that under the dark condition. Figure 4e shows that the temperature difference and open-circuit voltages (V_oc_) depend linearly on the laser power. This behavior indicates that the V_oc_ is primarily affected by the temperature difference, which is caused due to light absorption. The photovoltage due to the photothermoelectric effect is expressed as

$${V}_{PTE}=\left({S}_{2}-{S}_{1}\right)\Delta T,$$

where S_1_ and S_2_ are the Seebeck coefficients of the materials, expressed as S = V/∇T^23,24^. Based on the measured voltage differences with respect to the temperature differences in Fig. 4f, the Seebeck coefficient of the device was 7.9 μV/K, which was obtained via the linear fitting method. As the layer composed of graphene and gold primarily contributed to the temperature gradients, the Seebeck coefficient of the graphene can be expressed as follows: S_G_ (μV/K) = 7.9 (μV/K) + S_Au_ (μV/K), where S_Au_ has been reported to be approximately 1.9 μV/K near room temperature^28^. Therefore, the Seebeck coefficient of the graphene was approximately 9.8 μV/K, which is consistent with the values reported in literature (9 ~ 12 μV/K^29,30^).

## Conclusions

In summary, we demonstrated a Cr/SiO_2_/Cr broadband absorber on PET and Si substrates to compare the temperature differences caused by the difference in thermal conductivity. Based on the thermal images, the temperature differences were 160 K for the Cr/SiO_2_/Cr absorber on a PET substrate and 4 K for that on the Si substrate, under a 906-nm laser illumination with a laser power of 70 mW. The results of the thermal relaxation time indicated that the temperature differences were primarily affected by the thermal conductance of the substrates. The temperature differences of the Cr/SiO_2_/Cr absorber on the PET and Si substrates indicated the equal importance of thermal conductivity and absorbance to achieve high photothermal performance of the light absorber. Additionally, based on the temperature difference of the Cr/SiO_2_/Cr broadband absorber, the Seebeck coefficient of the monolayer graphene was found to be approximately 9.8 μV/K, which was obtained using the photothermoelectric effect. The result of the photothermoelectric effect indicated that the Cr/SiO_2_/Cr broadband absorber with the PET substrate can be used as an absorber in photothermoelectric devices including flexible applications, photothermal energy generators, and photothermal sensors.

## Methods

### Calculation

The reflection and transmission coefficients were calculated using the finite element method simulations. We used periodic boundary conditions with a unit cell of 100 nm along the x- and y-directions and perfectly matched layers at the boundaries in the z-direction. Plane waves were launched along the normal direction of incidence into the unit cell along the z-direction, and a power monitor was placed behind the source and the structure. The frequency-dependent complex refractive index of the material data, including Cr, SiO_2,_ and other materials used in the numerical simulations, was obtained from the data reported by Palik^31^.

### Preparation of the absorber

The Cr and SiO_2_ layers were simultaneously deposited on the PET and Si substrates, respectively, using an electron beam evaporator under high vacuum (~ 10^–7^ torr). Single-layer graphene was grown via chemical vapor deposition and subsequently transferred onto the Cr/SiO_2_/Cr structure via wet transfer.

### Measurements

The reflectance and transmittance were measured using a UV–VIS–NIR spectrophotometer (V-670, JASCO) with an integrating sphere from 400 to 1,500 nm at room temperature. The thermal images were captured using temperature measurement microscope systems (InfraScope III, Quantum Focus Instruments Corporation). The electrical measurements were performed using Agilent 4156C.
